# Editorial: Structural bioinformatics and biophysical approaches for understanding the plant responses to biotic and abiotic stresses

**DOI:** 10.3389/fpls.2022.1012584

**Published:** 2022-09-09

**Authors:** Raul A. Sperotto, Maria Hrmova, Steffen P. Graether, Luis Fernando S. M. Timmers

**Affiliations:** ^1^Graduate Program in Biotechnology, University of Taquari Valley – Univates, Lajeado, Brazil; ^2^Graduate Program in Plant Physiology, Federal University of Pelotas, Pelotas, Brazil; ^3^Faculty of Sciences, Engineering and Technology, University of Adelaide, Adelaide, SA, Australia; ^4^School of Life Science, Huaiyin Normal University, Huaian, China; ^5^Department of Molecular and Cellular Biology, University of Guelph, Guelph, ON, Canada; ^6^Graduate Program in Medical Sciences, University of Taquari Valley – Univates, Lajeado, Brazil

**Keywords:** bioinformatics and phylogeny, molecular dynamics, protein structural modeling and docking, plant defense, protein crystallization

Plants are exposed to a variety of environmental conditions that negatively impact their physiology and yields (Rivero et al., 2022; Sharma et al., 2022; Zandalinas and Mittler, 2022). Several studies identified mechanisms involving genes, proteins, and metabolites that underlie plant responses to stress conditions (Chen et al., 2022; Hassan et al., 2022; Huang et al., 2022; Mittler et al., 2022; Yang et al., 2022; Zhan et al., 2022). Some of these molecules were used to improve plant responses to abiotic and biotic stresses (Li et al., 2022; Mahto et al., 2022; Wang and Komatsu, 2022; Zhao et al., 2022). However, the underlying structural and functional relationships of these molecular mechanisms require more research. Computational and biophysical approaches are viable options for analyses of target molecules to understand their interactions and dynamics that initiate biochemical and physiological responses of plants (Wan et al., 2015; Konda et al., 2018; Moffett and Shukla, 2018; Rayevsky et al., 2019; Jha et al., 2022). These responses in turn control plant tolerance and resistance to sub-optimal environmental conditions. Therefore, this Research Topic is aligned with the current research trends and provides an update on the advances in structural bioinformatics and biophysical approaches to understanding plant responses to biotic and abiotic stresses at the molecular level. Here, we highlight some of the topics from the following contributions.

Biotic stress in plants involves the complex regulatory networks of various sensory/signaling molecules (Tiwari et al., 2022), including proteins that participate in plant defense strategies. Three articles focused on the genetic characterization of proteins involved in biotic stress resistance. First, Zheng et al. identified protein-protein interactions (PPIs) between rice and the fungus *Magnaporthe oryzae*, a causative agent of rice blast, which is the most devastating disease affecting rice production. A global PPI network consisted of 2,018 interacting protein pairs involving 1,344 rice proteins, where 418 blast fungus proteins showed enrichment for blast resistance genes—these network-based predictions would now allow discoveries of blast resistance genes in rice. Next, Sati et al. investigated computationally the interaction of the PsoR protein of plant-beneficial *Pseudomonas* spp. with various root exudates to better understand inter-kingdom signaling between plants and plant growth-promoting rhizobacteria (PGPR). The PsoR protein produces a range of antifungal and insecticidal secondary metabolites, making them useful biocontrol agents and thus helping during plant growth. A total of 59 different low molecular mass phytochemicals were virtually screened with the PsoR protein by molecular docking. Two root exudates, saponarin and 2-benzoxazolinone (BOA), present in the root exudates of barley and wheat, respectively, showed suitable binding with PsoR, likely showing cross-kingdom interactions. Lastly, Yadav et al. performed a wide-range analysis of Ca^2+^-sensing plant-specific calmodulin-like proteins (CMLs) in soybean to identify 41 true CMLs. Gene structural analysis and identifying conserved motifs and *cis*-acting elements in these targets strongly support their identity as the members of this family and involvement in stress responses. Further insights into the differential expression patterns of *GmCMLs* during *Spodoptera litura*-feeding, wounding, and signaling together with 3D structure prediction, identification of interacting domains, and docking of Ca^2+^ ions in *S. litura*-inducible GmCMLs provided evidence into their roles in Ca^2+^ signaling and plant defense during herbivory.

Two articles focused on the genetic characterization of proteins involved in abiotic stress tolerance and climate change and their impacts, suggesting potential candidates that could be targeted for plant breeding and genetic engineering. This is aimed at developing food crops that could thrive in deteriorating environmental conditions, and maintain or increase the crop yields (Ku et al., 2018). First, Arabia et al. outlined the role of the *Universal Stress Protein* (*USP*) gene family in rice (*Oryza sativa* L. ssp. *japonica*) to uncover 44 genes and their domain architecture that was key to the functional diversification under multi-stress environmental challenges. Next, Chen et al. described 33 *Brassica napus* L. *CONSTANS-LIKE* (*COL*) genes; these clustered into three subfamilies and exhibited conserved gene and protein structures, promoter motifs, and tissue-specific expression. The latter study also clarified the role of each gene subfamily during growth and development, flowering, and circadian rhythms.

Understanding of how bioactive molecules interact with their targets can help to explain their structure-activity relationships. In particular, the computational analyses of protein-ligand complexes can help to unravel these key interactions (Anighoro, 2020), providing valuable insights into the plant response to stress conditions. Two articles applied X-ray crystallography, comparative modeling, and molecular dynamics simulations to evaluate protein activity and protein-ligand interactions. In the first study, Infantes et al. outlined the importance of a single amino acid residue variation (PYR/PYL/RCAR) in ABA receptors, which promotes the transition of the latch and gate loops to active conformation in ABA-dependent and independent modes. Furthermore, the authors identified that the niacin molecule can act as an *in vivo* antagonist of ABA. In the second work, Maia et al. investigated the enzyme specificities of PR-4 SUGARWINs in sugarcane defense against phytopathogens. Based on experimental and computational methods, the authors showed the crystal structure of SUGARWIN2, the first PR-4 protein lacking an RNase activity. In addition, the authors suggested that SUGARWIN2 is more relevant to sugarcane defense against pathogenic fungi, as SUGARWIN2 showed higher expression levels after the *Colletothricum falcatum* (Went) treatment compared to the expression of SUGARWIN1.

Two additional articles focused on lipid barriers, which play critical roles in plant biology by separating spaces and generating compartments, and as biological barriers protect cells and organelles from stress conditions (Guo et al., 2019). In the review by Murray and Graether, these authors looked at the biophysical mechanisms by which dehydrins, a group of plant abiotic stress proteins, protect plants from cold damage. Despite dehydrin's lack of structure and low sequence conservation, the lysine-rich K-segments stabilized model membranes by maintaining fluidity and preventing membrane fusion. The paper by Ingram et al. examined a very different kind of lipid barrier—nanobubbles. The authors characterized the recently discovered 10–1,000 nm bubbles surrounded by a lipid monolayer, using molecular dynamics simulations. They show that they were stable and avoided embolisms at radii of 35 nm and under pressures of 0 to −1.5 MPa. To reconcile the presence of nanobubbles with the water transpiration model, the authors proposed that nanobubbles continuously expand and collapse into smaller bubbles, and reuse the lipids.

## Final comment

In summary, the work presented in this Research Topic documents how structural bioinformatics and computational biophysical approaches could be effectively applied to study the structure-function relationships of plant proteins and the function of small molecules involved in biotic and abiotic stresses. These molecules are at the core of the plant defense mechanisms that allow plants to mitigate stresses and enhance plant abilities to respond to unfavorable environmental conditions.

## Author contributions

All authors listed have made a substantial, direct, and intellectual contribution to the work and approved it for publication.

## Conflict of interest

The authors declare that the research was conducted in the absence of any commercial or financial relationships that could be construed as a potential conflict of interest.

## Publisher's note

All claims expressed in this article are solely those of the authors and do not necessarily represent those of their affiliated organizations, or those of the publisher, the editors and the reviewers. Any product that may be evaluated in this article, or claim that may be made by its manufacturer, is not guaranteed or endorsed by the publisher.
